# Supplementary material for: Consecutive treatments of methamphetamine promote the development of cardiac pathological symptoms in zebrafish
Source: PLoS One. 2023 Nov 17;18(11):e0294322. doi: 10.1371/journal.pone.0294322 (PMC10655962; doi:10.1371/journal.pone.0294322)
Supplement: S1 Fig — A repeated trial of both control and treatment fish was conducted, and averages are tabulated in the same format as shown in Fig 2. (A) Treatment fish displayed significantly decreased heart rate compared to control starting from day 3 of treatment (week 1). (B) Treatment fish exhibited a biphasic trend in heart rate variation (HRV), peaking at day 8 of treatment, corresponding to the beginning of week 2. (C) Treatment fish did not exhibit a significant difference in PR interval. (D) Treatment fish displayed a significant decrease in QTc during the end of week 1 and throughout week 2. (E) Treatment fish did not display a significant difference in QRS duration. * denotes p<0.05. (DOCX) [file pone.0294322.s001.docx]

Consecutive treatments of methamphetamine promote the development of cardiac pathological symptoms in zebrafish

(Supplemental Information)

Jimmy Zhang^1^, Anh H. Nguyen^2,3^, Daniel Jilani^2^, Ramses Seferino Trigo Torres^1^, Lauren Schmiess-Heine^2^, Tai Le^1^, Xing Xia^2^, Hung Cao^1,2,3^*

^1^Department of Biomedical Engineering, University of California-Irvine, Irvine, CA, USA.

^2^Department of Electrical Engineering and Computer Science, University of California-Irvine, Irvine, CA, USA.

^3^Sensoriis, Inc, Edmonds, WA, USA.

* Corresponding author

Email: hungcao@uci.edu (HC)


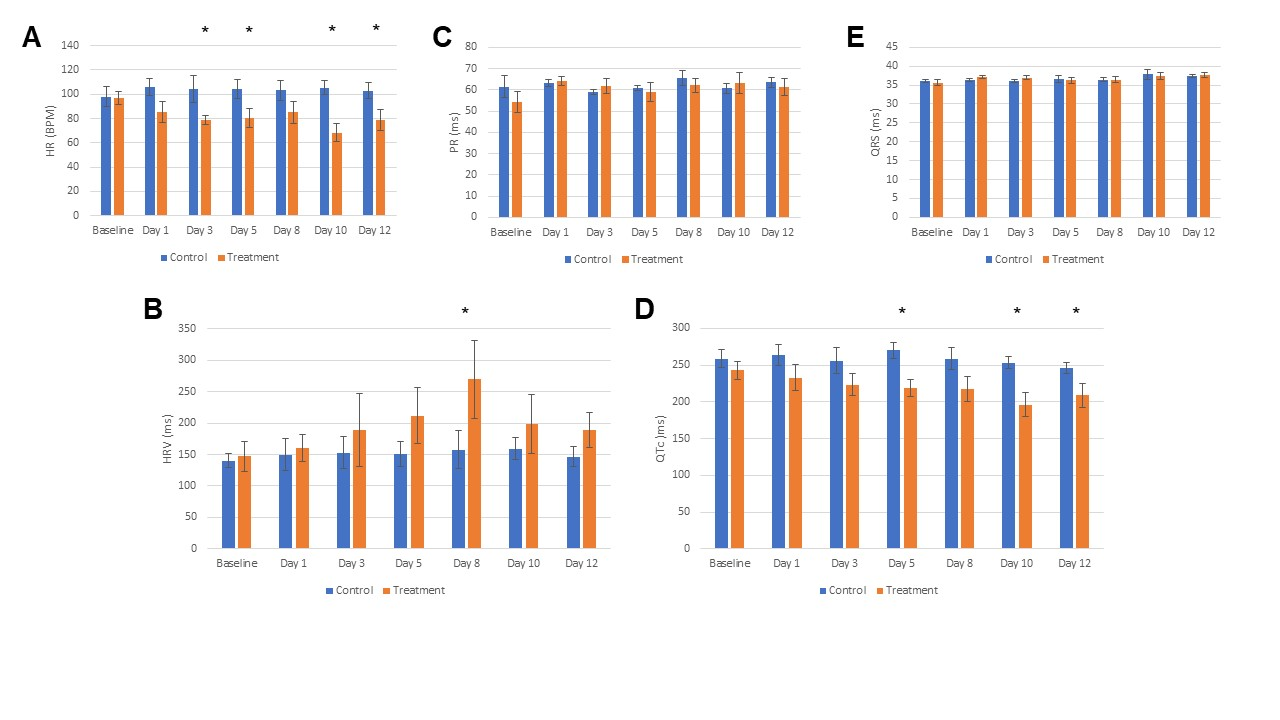


**S1 Fig. Second Trial of Electrophysiological Analysis of Meth Treatment Yields Similar Results.** A repeated trial of both control and treatment fish was conducted, and averages are tabulated in the same format as shown in Fig 2. (A) Treatment fish displayed significantly decreased heart rate compared to control starting from day 3 of treatment (week 1). (B) Treatment fish exhibited a biphasic trend in heart rate variation (HRV), peaking at day 8 of treatment, corresponding to the beginning of week 2. (C) Treatment fish did not exhibit a significant difference in PR interval. (D) Treatment fish displayed a significant decrease in QTc during the end of week 1 and throughout week 2. (E) Treatment fish did not display a significant difference in QRS duration. * denotes p<0.05.
