# Supplementary material for: Consecutive treatments of methamphetamine promote the development of cardiac pathological symptoms in zebrafish
Source: PLoS One. 2023 Nov 17;18(11):e0294322. doi: 10.1371/journal.pone.0294322 (PMC10655962; doi:10.1371/journal.pone.0294322)
Supplement: Attachment — Submitted filename: Response to Reviewers.pdf [file pone.0294322.s003.pdf]

Original Article Title: "*Consecutive treatments of methamphetamine promote the development of cardiac pathological symptoms in zebrafish*"

To: PLOS ONE Editors

Re: Response to reviewers

Dear Editors,

We appreciate your valuable comments and suggestions. We have carefully revised our manuscript following your comments point by point. The revisions are highlighted by mark-ups found within the file labeled 'Revised Manuscript with Track Changes'. Your constructive comments are extremely helpful for us to improve our work as well as to better revise the current manuscript.

Please find our detailed answers to reviewers' comments beginning from the next page.

Best regards,

J. Zhang *et al.*
Department of Biomedical Engineering
University of California, Irvine

Reviewer #1: Review Comments

This report by Zhang and colleagues aims to elucidate the effect of methamphetamine (Meth) on cardiac physiology and electrophysiology in a standardized and controlled setting. To this end, the authors exposed adult zebrafish to meth and measured cardiac function using electrocardiography. This analysis suggests that treatment with Meth induces electrical alterations in the zebrafish heart, which the authors suggest are associated with changes in cAMP in cardiomyocytes.

While we acknowledge the authors' hard work, several weaknesses in the experimental design and the interpretation of the results enormously reduce our enthusiasm. In short, the authors fail to justify their experimental setup, present minimal data and lack the required results to support most of their claims. For example, the authors claim that their goal is to "elucidate the mechanisms by which Meth induces electrophysiological abnormalities," but they only explored changes in cAMP. Moreover, the results are poorly integrated with the text, which would benefit from extensive English revisions and editing. The following criticisms do not cover all the detected issues and should not be considered an exhaustive list. However, it might be a good starting point for the authors to revisit their results if they decide to resubmit their manuscript elsewhere.

MAJOR COMMENTS:

(1) Overstatements and unjustified claims. The main problem of this manuscript is the lack of data to support most of the claims. Indeed, the experimental data presented here is rather meager. Illustrative examples:

Line 331: "[we have sought to] Elucidate the mechanism of Meth in inducing electrophysiological abnormalities." This study does not elucidate this. It characterizes some of the electrophysiological abnormalities observed after meth intake, but does not resolve how they have been generated. Showing an increase of cAMP signaling after meth treatment is not sufficient to make that claim. In this study, they show it is correlative, they don't demonstrate that it is causative.

Author response: Thank you for pointing this out! We have rephrased the aforementioned statement to "[we have sought to] characterize the electrophysiological abnormalities due to meth administration in a controlled environment."

Line 355-356: "To the best of our knowledge, this is the first time sinus block has been indicated as a symptom of Meth use." This has never been demonstrated.

Author response: Thank you for the comment! We have removed the statement in question and replaced it with "ECG acquired from Meth-treated fish displayed episodes of sinus arrest, where an instance of cardiac conduction normally present in regular sinus rhythm is absent."

Line 425: "We propose that cAMP upregulation may be regulated by GPCRs such as TAAR1, as depicted in Fig 5A." The data presented in this manuscript do not support this model though.

Author response: Thank you for the valuable feedback! We have conducted additional experiments, including the production of TAAR1-overexpressed HEK293 cells for cAMP and Ca^{2+} analysis. We have also utilized EPPTB, a selective TAAR1 antagonist, as an inhibitor to illustrate the integral role of TAAR1 in downstream dysregulation. The new data can be found in Fig 3.

Line 432-433: "The results in this study also indicated that the upregulation of cAMP occurred in a dose-dependent manner and can be inhibited by an antagonist, displaying the plausible reversibility of Meth-induced cardiotoxicity". This conclusion cannot be drawn from the presented results.

Author response: Thank you for the comment! The statement has been modified to "Using the GloSensor cAMP assay, we demonstrated that the upregulation of cAMP occurred in a dose-dependent manner within zebrafish cardiomyocytes. This upregulation was inhibited by an antagonist of cAMP." Notably, we removed the statement on the reversibility on Meth-induced cardiotoxicity.

(2) Experimental Design and Interpretation. Another important problem is the apparently arbitrary experimental design, including the lack of appropriate controls. The authors are treating animals with Meth, and then measuring defects in electrical activity and cAMP, but the characterization is incredibly superficial. Specific examples:

• Is this set-up standard to do electrophysiological recording? Why the need to come up with their own?

Author response: Thank you for the questions! The use of micro-needle electrodes for zebrafish ECG recording was first developed in the PI's previous lab [1]. Our lab continues on this line of research to improve the set-up for various cardiovascular applications. The set-up described in the current manuscript was designed to facilitate easier and faster manipulation of the electrodes. This feature was vital for conducting ECG measurements for this study and future drug testing studies in a feasible manner.

• Please justify treatment concentrations and durations: please add justification on the dose of Meth used: why 200 μm? Why 20 min of treatment at 200 μm? Why 3 times a week? Does that correlate somehow with drug intake in humans? Why quantification only for two weeks?

Author response: Thank you for your questions! The treatment regimen of twenty minutes and the concentration for Meth were first determined from a previous zebrafish Meth study [2]. Prior to the study described by our manuscript, we conducted a pilot study to determine the suitable concentration for reducing mortality and maximizing significance. We proceeded with 200 μM as the experimental concentration. We treated the zebrafish 3 times a week in order to allow the zebrafish at least one day to recover from measurement, as tricaine, the anesthetic agent, has been known to affect ECG parameters due to its effect to block sodium channels [3]. As there were no previous studies analyzing Meth-associated cardiotoxicity in zebrafish, we hope that the study

detailed in our manuscript will provide a basis for future Meth studies. The duration of two weeks was initially set based on previous long-term Meth-associated cardiotoxicity studies [4, 5].

• Figure 5: HEK293 used as control to test for cell specificity. I am not sure this is the most appropriate control. Perhaps a better one to claim that meth specifically induces cardiotoxicity would have been to isolate other cells types from treated fish and do the same quantification.

Author response: Thank you for the suggestion! While we have considered using other cell types as a control for cAMP expression, we are currently not able to isolate those cell types in a reliable manner. As an alternative, we developed a HEK cell line with overexpressed zebrafish TAAR1 as a positive control.

• Not clear how the dose-dependent experiments have been performed in Figure 5C-D: same cells subjected to different increasing doses, or different cells?

Author response: Thank you for the question! The original cell population isolated from the zebrafish heart was separated into the respective groups for treatment with different increasing doses.

1) Rigor and Reproducibility of the Data.

• In figure 4: what would be the explanation for the drop of HR also in untreated fish at day 8 compared to other days (which looks significant to other days in untreated fish)?

Author response: Thank you for the question! The explanation for the drop of HR between days 5 and 8 is the consistent exposure to tricaine. As an anesthetic agent, tricaine has been known to decrease muscle contractility and heart rate [6]. Repeated tricaine treatment for zebrafish induced increased susceptibility to the effects of tricaine, as shown in a previous study [7]. This susceptibility may be varied between individual organisms. As shown in the second iteration of our experiment, the heart rate in untreated fish did not display a noticeable decline throughout the course of the experiment. However, the conclusion of our study delineates that Meth-treated fish exhibited a greater decrease in heart rate than untreated fish, accounting for tricaine effects for both experimental groups.

• Figure 4B: For this quantification, it looks like the HRV is actually also increasing after 10 days in untreated fish. Please comment.

Author response: Thank you for the insight! The slow increase in HRV in untreated fish near the conclusion of the experiment may be due to the presence of arrhythmias due to tricaine treatment, which increased the variation in heart rate. It has been established that anesthetic agents cause arrhythmic instances [8]. In the second trial, we determined that HRV did not increase in untreated fish. The conclusion drawn from our results indicates that Meth induces additional arrhythmias

during the first stage of our study, followed by a decrease in HRV due to the significant decrease in heart rate overtaking the presence of arrhythmias.

• Figure 4C: For this result, it is worth noting that based on the quantification, it actually looks like the PR stayed pretty constant in Meth-treated fish (~55ms), and that the differences between the two experimental groups seem to actually be carried by variations of the PR in the untreated ones along the course of the experiment (ranging from ~55 up to 69 ms). Explanations?

Author response: The variation in the PR interval may be due to the effects of anesthesia, as the agent slows heart conduction. In our second trial, we determined that PR interval did not significantly change between the untreated and treated groups, and the discussion has been modified accordingly. We would like to emphasize that the major conclusions of this manuscript include the progressive decrease in heart rate and the differences seen in HRV. The differences in PR interval were determined to be significant only in the early stage of treatment, and the explanation for this observation is most likely the antagonistic effects of anesthesia and Meth.

MINOR COMMENTS:

Figure 1-3 should be merged. They have only two panels each (A,B). Poorly integrated and discussed in the text. Some conclusions only described in figure legends.

Author response: Thank you for the suggestion! Figures 1-3 have been merged as a single figure. Discussions and conclusions derived from the figure have been included in the Results and Discussion sections.

Legend Figure 4: (C) Meth treatment induced a significant decrease during week 1 but not week 2 of treatment. Please specify what is decreasing in the legend.

In figure 5, forskolin is written with an e at the end, please correct.

Please specify somewhere in the manuscript what forskolin does.

Author response: Thanks for pointing this out! The aforementioned comments were addressed in the respective figures (Figs 2 and 3) and in the 'GloSensor cAMP assay' subsection under 'Methods'. We have replaced forskolin with TAAR1-overexpressed HEK293 cells as the positive control.

Not clear why the potential effect of tricaine is being brought up in the discussion when the authors do not address that at all experimentally.

Author response: Thank you for the comment! We realized that we did not fully clarify the potential confounding effects of tricaine (anesthetic agent) on the ECG data we have presented in the study.

We have further elaborated on the limitations of using anesthesia for ECG acquisition in our discussion, with some discussed in previous comments regarding the data in Figure 4.

Line 32, in abstract: replace “previous” with “previously”.

Line 33, in abstract: typo: replace “has” with “have”, as assays plural.

Line 37, in abstract: replace “and remain”, with “that remained”.

Line 45, in abstract: add s to increases, as Methamphetamine is singular.

Line 91: Awkward phrasing: “it necessitates the research to”

Line 98: replace “its” by “their”

Line 103: “of” to be replaced by “to”?

Line 103: specify contractility of what; cell? Cardiomyocytes?

Line 110: might be worth specifying the remodeling of what.

Line 115: add “to” after “due”

Line 120: Similarity to what? to the study ref24? If not, please specify similarity to what. If when, it might add clarity to the sentence to replace "used for" to "to".

Line 143-144: Awkward redundancy, fix?

Line 179: Please add “be” after “can” to have “can be accessed”.

Line 234: I think “desiderate” should be replaced by “desiderated” in this sentence

Line 234: GloSensor instead of Glosensor for consistency

Author response: Thank you for your thorough revisions! We have implemented the specified changes in the respective sections.

Line 92-94: Quite the statement. I am sure a lot of non-zebrafish people would argue that it is not essential to understand meth abuse to fish to understand meth-induced cardiac toxicity.

Author response: Thank you for your comment! We have revised the statement to “Therefore, better understanding of the cardiac dynamics of Meth abuse in zebrafish, a relevant model for human cardiac studies, may be vital for the future Meth-associated research.”

Line 124: They did not really look at GPCRs per se though.

Author response: Thank you for your comment! We have conducted additional assays on the proposed GPCR pathway within our study. The statement was revised to “We then sought to explain the results by conducting molecular analysis of the GPCR pathway within heart tissue, including the effect on fibrotic and Ca2+ dysregulation, which attribute to cardiac toxicity.”

Line 133: Might be worth specifying the genetic background of WT.

Author response: Thank you for your comment! The zebrafish we used in our study is of the AB strain of WT zebrafish.

Line 249: What is the maximum effect expected in this experimental setting?

Author response: Thank you for the question! The maximum effect obtained in the cAMP and calcium assays were approximately 1400000 RLU and 12000 RFU, respectively.

Line 258: Number of animals quite low for this study (CTL= 6; treated= 8).

Author response: Thank you for pointing this out! We have conducted a second trial of ECG measurements and included the new results in the most recent draft.

Reviewer #2: Review Comments

In this manuscript the authors describe a preparation and a study in which they record cardiac activity from adult zebrafish following methamphetamine treatment. The goal of the work was to examine the relationship between cardiotoxicity and arrhythmia with associated methamphetamine exposure in adult zebrafish. Animals were treated with Meth 3 times a week for 2 weeks and multiple ECG measurements were taken. The authors also examined cAMP levels via a cAMP assay to ascertain the effect on this second messenger following Meth treatment.

I am reasonably convinced that meth acts either directly or indirectly on cardiac activity, but I am more skeptical about the mechanism and the nature of the interactions. The authors have not provided convincing evidence that zebrafish either have TAAR1 and that it is expressed in cardiac tissue (PCR or immunohistochemistry if antibodies exist). I understand that antagonists for TAAR1 are limited but a compound such as EPPTB which (I believe) is an inverse agonist might provide some information on the actions of Meth on TAAR1 in this study. I have also found a number of grammatical and spelling errors throughout the manuscript and respectfully suggest that the authors pay particular attention to these details.

Additional comments are listed below.

1. Abstract, Line 31-32: the sentence that reads "zebrafish underwent electrocardiogram (ECG)…" needs to be adjusted because many readers may not know what the previously developed acquisition system is. In other words, clarify what the authors are referring to.

Author response: Thank you for pointing this out! The sentence was revised to "…zebrafish underwent electrocardiogram (ECG) measurement using an in-house developed acquisition system for electrophysiological analysis." This clarifies that the system is based on previous work conducted in our lab, which is referenced in the introduction.

2. Abstract, line 36-38: Should probably read "…exhibited a decrease in heart rate at the end of the first week of treatment, which remained significantly lower than untreated fish in the second week.

6. Methods, line 169: This sentence sounds a bit odd. Please review and correct if necessary.

3. Introduction, line 97-98: It may be how I read the sentence but to me this sentence implies that all GPCRs use cAMP as a second messenger. Please adjust for accuracy.

12. Figure 5: Methamphetamine, Forskolin and Arrhythmia are misspelled. Please double check all spelling.

Author response: Thank you for the comments. We have revised the corresponding sentences for accuracy and clarity.

4. What is known about TAAR1 in zebrafish? can the authors provide evidence (maybe a reference or PCR etc) for TAAR1 expression in zebrafish?

Author response: Thank you for your question! Research regarding TAAR1 expression in zebrafish has mainly been conducted for the neurological system, specifically, the olfactory system [9]. Similar neurological studies in other animal models have been published. However, the

understanding of how TAAR1 expression may influence cardiovascular pathology remains poorly understood, even though TAAR1 has been discovered to be expressed in the heart [10]. Given that Meth triggers cAMP via TAAR1 expression, it would be beneficial to explore Meth-induced TAAR1 expression and modulation for future cardiovascular studies. We have produced TAAR1-overexpressed HEK293 cells to demonstrate that Meth upregulates TAAR1 and causes downstream dysregulation, as seen in Fig 3.

5. Methods, line 155: I think this is the first time PDMS is used. Please explain what this is.

Author response: Thank you for the question! PDMS, or polydimethylsiloxane, is a flexible and biocompatible polymer most commonly used in biosensors and implants. Therefore, it serves as a suitable material for housing zebrafish.

7. Methods, line 219: Please explain what is BDM?

Author response: Thank you for asking! BDM, or butanedione monoxime, is a contraction inhibitor for cardiomyocytes. Used by Sander *et al.* for zebrafish cardiomyocyte isolation, BDM was most likely utilized to increase viability of the cardiomyocytes [11].

8. Methods line 241: Please explain what is the JMP suite?

Author response: Thanks for asking! The JMP suite is a statistical analysis and data visualization software.

9. Results: I found little indication of numbers of experiments and animals throughout the Results section or in the Figure legends.

Author response: Thank you for the comment! We have included the number of animals used (control, n=6; treatment, n=8) as well as the number of trials (2) in the relevant sections in Results and Figures.

10. Acknowledgements: It seems odd to me to Acknowledge persons who are already listed as Authors of the manuscript.

Author response: Thank you for pointing this out. The corresponding section has been removed.

11. Figure 4: Apologies if I missed this but can the authors Discuss why there are some effects at intermediate time periods (eg Fig 4C Day 5) but not at earlier or later time periods (Day 3 or Day 8, 10).

Author response: Thank you for the question! In the case of heart rate and QTc, significance was not seen in the early time periods most likely because the accumulative effect of consistent Meth intake had not manifested. This effect was first seen from day 5. The lack of significance seen in day 8 may be due to the effects of anesthesia, which also shifted the value for untreated fish, although not to the same degree as those in treated fish. Anesthesia is known to slow heart rate and consequently decrease QTc. For HRV, it did not display significance for before and after day 5 due to the counteracting effects of arrhythmic symptoms, which increased HRV, and cardiac damage, which was displayed by decreased HRV. This cardiac damage is consistent with the results found in cardiomyopathy due to Meth intake [12-14]. For PR interval, the significance seen in days 1 and 5 was due to the differences induced in untreated fish group. This effect may be caused by anesthesia, which increased PR interval. However, it is notable that for the second trial, the days of significance are different, such as HRV peaking on day 8 and PR interval not displaying significance. These variations were most likely due to varying pharmacokinetic responses to Meth and anesthesia.

Journal Requirements:

1. Please ensure that your manuscript meets PLOS ONE's style requirements, including those for file naming. The PLOS ONE style templates can be found at

https://journals.plos.org/plosone/s/file?id=wjVg/PLOSOne_formatting_sample_main_body.pdf and

https://journals.plos.org/plosone/s/file?id=ba62/PLOSOne_formatting_sample_title_authors_affiliations.pdf

Author response: Thank you for the reminder! The revised manuscript has been reformatted to PLOS ONE style.

2. To comply with PLOS ONE submissions requirements, in your Methods section, please provide additional information regarding the experiments involving animals and ensure you have included details on (1) methods of sacrifice, (2) methods of anesthesia and/or analgesia, and (3) efforts to alleviate suffering.

Author response: Thank you for the reminder! The methods section has been updated to include the method of anesthesia, euthanasia, and efforts to alleviate suffering. The additions were made under ‘Zebrafish husbandry and preliminary surgical procedures’ and ‘ECG recording procedure and instrumental setup’ subsections.

3. In your Data Availability statement, you have not specified where the minimal data set underlying the results described in your manuscript can be found. PLOS defines a study's minimal data set as the underlying data used to reach the conclusions drawn in the manuscript and any additional data required to replicate the reported study findings in their entirety. All PLOS journals require that the

minimal data set be made fully available. For more information about our data policy, please see http://journals.plos.org/plosone/s/data-availability.

"Upon re-submitting your revised manuscript, please upload your study's minimal underlying data set as either Supporting Information files or to a stable, public repository and include the relevant URLs, DOIs, or accession numbers within your revised cover letter. For a list of acceptable repositories, please see http://journals.plos.org/plosone/s/data-availability#loc-recommended-repositories. Any potentially identifying patient information must be fully anonymized.

Important: If there are ethical or legal restrictions to sharing your data publicly, please explain these restrictions in detail. Please see our guidelines for more information on what we consider unacceptable restrictions to publicly sharing data: http://journals.plos.org/plosone/s/data-availability#loc-unacceptable-data-access-restrictions. Note that it is not acceptable for the authors to be the sole named individuals responsible for ensuring data access.

We will update your Data Availability statement to reflect the information you provide in your cover letter.

Author response: Thank you for the reminder! We have included our Data Availability statement here and in the updated cover letter:

"[The authors] have also declared that all underlying data for the conclusions made in this manuscript are included in this manuscript."

4. Please amend the manuscript submission data (via Edit Submission) to include authors Dr. Lauren

Schmiess-Heine, Dr. Tai Le, and Dr. Xing Xia.

Author response: Thank you for the reminder! The authors have been included in the revision within the submission system.

5. Your ethics statement should only appear in the Methods section of your manuscript. If your ethics statement is written in any section besides the Methods, please delete it from any other section.

Author response: Thank you for pointing this out! We have removed the ethics statements that were located outside the Methods section.

References used in this letter

1. Yu F, Li R, Parks E, Takabe W, Hsiai TK. Electrocardiogram Signals to Assess Zebrafish Heart Regeneration: Implication of Long QT Intervals. Annals of Biomedical Engineering. 2010;38(7):2346-57. doi: 10.1007/s10439-010-9993-6.

2. Tackie-Yarboi E, Wisner A, Horton A, Chau TQT, Reigle J, Funk AJ, et al. Combining Neurobehavioral Analysis and In Vivo Photoaffinity Labeling to Understand Protein Targets of Methamphetamine in Casper Zebrafish. ACS Chemical Neuroscience. 2020;11(17):2761-73. doi: 10.1021/acschemneuro.0c00416.
3. Attili S, Hughes SM. Anaesthetic tricaine acts preferentially on neural voltage-gated sodium channels and fails to block directly evoked muscle contraction. PLoS One. 2014;9(8):e103751. Epub 2014/08/05. doi: 10.1371/journal.pone.0103751. PubMed PMID: 25090007; PubMed Central PMCID: PMCPMC4121177.
4. Sun X, Wang Y, Xia B, Li Z, Dai J, Qiu P, et al. Methamphetamine produces cardiac damage and apoptosis by decreasing melusin. Toxicology and Applied Pharmacology. 2019;378:114543. doi: https://doi.org/10.1016/j.taap.2019.03.015.
5. Rorabaugh BR, Seeley SL, Stoops TS, D'Souza MS. Repeated exposure to methamphetamine induces sex-dependent hypersensitivity to ischemic injury in the adult rat heart. PLOS ONE. 2017;12(6):e0179129. doi: 10.1371/journal.pone.0179129.
6. Dziaman R, Kłyszejko B, Hajek G. The effect of MS-222 on the cardiac and respiratory function and behaviour of common carp, Cyprinus carpio L., during general anaesthesia. Acta Ichthyologica et Piscatoria. 2005;35(2):125-31.
7. Owen JP, Kelsh RN. A suitable anaesthetic protocol for metamorphic zebrafish. PLOS ONE. 2021;16(3):e0246504. doi: 10.1371/journal.pone.0246504.
8. Kwon CH, Kim SH. Intraoperative management of critical arrhythmia. Korean journal of anesthesiology. 2017;70(2):120-6. Epub 2017/04/04. doi: 10.4097/kjae.2017.70.2.120. PubMed PMID: 28367281; PubMed Central PMCID: PMCPMC5370296.
9. Hussain A, Saraiva LR, Korsching SI. Positive Darwinian selection and the birth of an olfactory receptor clade in teleosts. Proceedings of the National Academy of Sciences. 2009;106(11):4313-8. doi: 10.1073/pnas.0803229106.
10. Bunzow JR, Sonders MS, Arttamangkul S, Harrison LM, Zhang G, Quigley DI, et al. Amphetamine, 3,4-methylenedioxymethamphetamine, lysergic acid diethylamide, and metabolites of the catecholamine neurotransmitters are agonists of a rat trace amine receptor. Molecular pharmacology. 2001;60(6):1181-8. Epub 2001/11/28. doi: 10.1124/mol.60.6.1181. PubMed PMID: 11723224.
11. Sander V, Suñe G, Jopling C, Morera C, Belmonte JCI. Isolation and in vitro culture of primary cardiomyocytes from adult zebrafish hearts. Nature Protocols. 2013;8(4):800-9. doi: 10.1038/nprot.2013.041.
12. Bazmi E, Mousavi F, Giahchin L, Mokhtari T, Behnoush B. Cardiovascular Complications of Acute Amphetamine Abuse: Cross-sectional study. Sultan Qaboos University medical journal. 2017;17(1):e31-e7. Epub 2017/04/19. doi: 10.18295/squmj.2016.17.01.007. PubMed PMID: 28417026; PubMed Central PMCID: PMCPMC5380419.
13. Sinha A, Lewis OD, Kumar R, Yeruva SLH, Curry BH. Amphetamine Abuse Related Acute Myocardial Infarction. Case Reports in Cardiology. 2016;2016:7967851. doi: 10.1155/2016/7967851.
14. Wang Q, Michiue T, Ishikawa T, Zhu BL, Maeda H. Combined analyses of creatine kinase MB, cardiac troponin I and myoglobin in pericardial and cerebrospinal fluids to investigate myocardial and skeletal muscle injury in medicolegal autopsy cases. Legal medicine (Tokyo, Japan). 2011;13(5):226-32. Epub 2011/06/21. doi: 10.1016/j.legalmed.2011.05.002. PubMed PMID: 21683643.
